# The origins of babytalk: smiling, teaching or social convergence?

**DOI:** 10.1098/rsos.170306

**Published:** 2017-08-02

**Authors:** Marina Kalashnikova, Christopher Carignan, Denis Burnham

**Affiliations:** The MARCS Institute for Brain, Behaviour and Development, Western Sydney University, Locked Bag 1957, Penrith 2527, Australia

**Keywords:** infant-directed speech, electromagnetic articulography, vowel hyperarticulation

## Abstract

When addressing their young infants, parents systematically modify their speech. Such infant-directed speech (IDS) contains exaggerated vowel formants, which have been proposed to foster language development via articulation of more distinct speech sounds. Here, this assumption is rigorously tested using both acoustic *and*, for the first time, fine-grained articulatory measures. Mothers were recorded speaking to their infant and to another adult, and measures were taken of their acoustic vowel space, their tongue and lip movements and the length of their vocal tract. Results showed that infant- but not adult-directed speech contains acoustically exaggerated vowels, and these are not the product of adjustments to tongue or to lip movements. Rather, they are the product of a shortened vocal tract due to a raised larynx, which can be ascribed to speakers' unconscious effort to appear smaller and more non-threatening to the young infant. This adjustment in IDS may be a vestige of early mother–infant interactions, which had as its primary purpose the transmission of non-aggressiveness and/or a primitive manifestation of pre-linguistic vocal social convergence of the mother to her infant. With the advent of human language, this vestige then acquired a secondary purpose—facilitating language acquisition via the serendipitously exaggerated vowels.

## 1. Background

When parents interact with their young infants, they unconsciously produce a distinct speech register, babytalk, more formally known as infant-directed speech (IDS). In comparison to adult-directed speech (ADS), IDS has simplified grammatical structure [1], longer pauses, higher mean pitch and greater pitch range [2,3], heightened positive affect [4], more distinguishable vowels [5,6] and distinct facial features [7]. Evidence showing that IDS contains acoustically exaggerated speech (ES) sounds [5,6] has been taken to demonstrate that IDS is a powerful tool that parents use to facilitate linguistic development [8]. In this study, we evaluate this linguistic function hypothesis by examining the possible articulatory sources of acoustically exaggerated vowels in IDS: lip movements, tongue movements and vocal tract length, or some combination of these.

The linguistic function hypothesis rests on the finding that articulation of IDS vowels is acoustically exaggerated, resulting in more distinct phonetic categories and thus more intelligible speech [9]. This vowel hyperarticulation is indexed by the area of the triangle that results from plotting first and second formant (F_1_, F_2_) values of the three corner vowels—/i/, /u/, /a/ (the vowel sounds ‘ee’, ‘oo’, ‘ah’)—in two-dimensional space. If these corner vowels are produced closer to the peripheral limits of a speaker's vowel space, then the two-dimensional F_1_/F_2_ triangle area increases, thus enhancing the perceptual distance between the three vowels. Using this method, vowel triangles in IDS have indeed been found to be significantly larger than in ADS [5,6].

As further evidence of a linguistic function in IDS, vowel hyperarticulation is also present in speech addressed to other audiences with linguistic potential, such as foreigners [10], computer avatars [11] and parrots [12], but absent when the audience lacks linguistic potential, as in the case of speech to dogs and cats [5]. Furthermore, vowel hyperarticulation is absent in IDS addressed to infants whose auditory processing ability is compromised due to a sensory [13,14] or cognitive [15] impairment. These results from other audiences and from infants with different receptive abilities suggest that the linguistic function of IDS is additionally a didactic function: parents, albeit unconsciously, only engage hyperarticulated vowels in IDS when the infant is able to benefit from it, and abandon it for infants with sensory or cognitive impairments, perhaps in favour of other potentially more effective adaptations to capture and maintain infants' attention.

More peripheral formants and thus exaggerated vowels can originate from modulations at three articulatory loci: shortened vocal tract, exaggerated lip movements and exaggerated tongue movements. The first potential articulatory locus, shorter vocal tract, can result from either retracting the lips or raising the larynx, or from both. The action of raising the larynx rotates the cricoid cartilage, creating tension in the vocal folds [16], thus increasing pitch.^1^ A shortened vocal tract also results in higher formant frequencies and greater spacing between formants. So when the larynx is raised, there is both an increase in pitch and an expansion of the vowel triangle, which happen to be two of the most prominent characteristics of IDS [3,5,6]. Across species a shorter vocal tract is generally characteristic of a smaller body type and, as many non-human species raise their larynx in offspring-directed vocalizations, this adaptation has been suggested to function as a signal of non-aggression and friendliness [17]. Thus, this social-emotional purpose is attributed to vocal tract shortening, which is independent of the articulatory posture of the tongue and the lips (i.e. the active articulators involved in modifying vowel quality) and is not unique to human vocalizations. This positions it as an adjustment that is not aimed at achieving the linguistic or didactic purpose attributed to vowel hyperarticulation. Thus, it is possible that human adults employ this same articulatory adjustment to appear smaller, friendlier and less threatening when addressing their infants. *If this were the case, then the vocal tract in IDS should be shorter than in ADS.*

The second potential articulatory locus for exaggerated vowels is lip movements. Smiling, which occurs frequently in IDS [18] involves mouth opening, lip spreading and retraction of the lips. These actions also shorten the vocal tract—albeit from the lip end of the vocal tract rather than from the larynx end—and it results in more extreme formant values but not in heightened pitch [19]. Exaggerated movements of the lips can also result in more distinct visual articulation of vowels. Indeed when viewing videos of mothers speaking to their young babies, naive raters identify three unique facial expressions [7]. Interestingly, these three expressions can be directly mapped to the lip movements when producing the three corner vowels: exaggerated lip spreading (mapping to the vowel /i/), exaggerated lip rounding/protrusion (mapping to the vowel /u/) and exaggerated lowering of the jaw and lip opening (mapping to the vowel /a/). Thus, it is possible that IDS corner vowels are visually more distinguishable than ADS vowels by virtue of their emotional connotations [20]. *If this were the case, then lip measures of protrusion and aperture should be more exaggerated in IDS than ADS.*

Each of the above two articulatory loci, larynx raising and lip exaggeration, imply that the exaggerated vowels in IDS are a side effect of articulatory adjustments unrelated to linguistic functions. The third potential articulatory locus, exaggerated tongue movements, does not imply that the exaggerated vowels in IDS are a side effect, rather that parents exaggerate vowels in IDS for a didactic purpose. According to this view, such articulatory exaggeration of tongue movements is proposed to be directed at producing more distinct and canonical phonetic categories that facilitate infants’ perceptual discrimination and reproduction of these categories in their own vocal tract. *If this is true, then over and above exaggerated facial expressions or shortening of the vocal tract (aimed to appear non-threatening), exaggerated tongue and lip movements* must *be present in IDS.*

Previous research has focused on acoustic measurements of vowel productions in IDS compared with ADS. This is the first study to collect objective articulatory measures for IDS and ADS in order to identify the source of the now well-established vowel hyperarticulation in IDS, rather than inferring information about articulation from acoustics. In this study, both articulatory and acoustic data were collected from mothers interacting with their 11-month-old infants and an adult experimenter. Each mother completed three recording sessions: IDS (interacting with her infant), ADS (interacting with a female experimenter) and ES in which the mother was asked to produce exaggerated versions of /i/, /u/ and /a/ without addressing an interlocutor. The three speech registers (IDS, ADS and ES) were compared for: hyper-acoustics, and three aspects of articulation—tongue movements, lip movements and vocal tract length.

For each articulatory locus, ES and ADS were compared first to determine whether articulatory exaggeration is possible at that locus. Second, IDS and ES were compared to assess whether IDS is produced at the articulatory limits of each locus. Finally, IDS and ADS were compared to determine whether IDS articulations are more exaggerated than in ADS.

## 2. Method

### 2.1. Participants

Eight mothers (*M* age = 35.4 years, s.d. = 4.8) and their 11-month-old infants (two female, *M* age = 48.7 weeks, s.d. = 2.8) participated in the study. All mothers were native speakers of Australian English. All infants were acquiring English in a monolingual context and were not at risk for sensory or cognitive impairment.

### 2.2. Procedure and apparatus

In the IDS session, the mother sat facing her infant who sat in a high chair. To elicit productions of the three corner vowels /i/, /a/, /u/, mothers were provided with a toy sheep, a toy shark and a baby shoe and asked to play with and name these objects frequently. In the ADS session, the mother sat facing the experimenter and completed a brief interview about the IDS play session, again frequently naming the three target words. In the ES session, the mother was instructed to produce each of the three target vowels five times according to the following instructions: ‘say the vowel “ah” as if you were really surprised or excited’, ‘say the vowel “ee” as if you were really happy’, and ‘say the vowel “oo” as if you were really concerned’. These three emotional terms have been previously attributed to the three predominant facial expressions that mothers employ when interacting with their young infants [7]. For the purposes of this study, we interpreted the production of these exaggerated vowel tokens as models of truly hyperarticulated speech productions. All mothers completed the sessions in the order IDS, ADS and ES. The mothers were informed that the study investigated their mouth movements while they interacted with their infant, but they were not aware of the focus on their vowel production.

Three-dimensional electromagnetic articulography (EMA) data were collected at 100 Hz using a Wave system from Northern Digital, Inc. EMA technology uses low-amplitude electrical signals generated in alternating-current electromagnetic fields and subsequently induced in small copper coil sensors placed within the fields. The relative strengths of the induced signals are used to monitor the position of these sensors over time. When the sensors are adhered to flesh points along the vocal tract (such as the tongue surface and around the lips), the changing position of these vocal tract flesh points can be tracked with high spatio-temporal resolution in a safe, non-invasive manner.

Three sensors were adhered along the central midline of the tongue: at the tongue tip (TT; approx. 5 mm from the end of the tongue tip), at the tongue back/dorsum (TB; as far back as could be reached by the experimenter without discomfort to the participant) and at the middle of the tongue (TM; placed equidistant between TT and TB). Four additional sensors were adhered around the lips: at the vermillion border of the upper and lower lips, and at the left and right corners of the mouth. Three sensors were attached to physically stable locations around the periphery of the head: the mastoid processes behind the left and right ears, and near the gum line at a point between the superior incisors. Since these locations do not move relative to the head during the experiment, the spatial data from these sensors were used to correct for any head movements made during the experiment, so that the movement of the tongue and lip sensors can be interpreted as movement relative to each mother's head space. Finally, three sensors adhered to a plastic protractor were used to rotate and translate the EMA data to a similar origin for all of the mothers, so that the data could be combined and collapsed across speakers and projected from a comparable origin. At the end of each experimental session, the mother was instructed to bite down on this protractor and the experimenter adjusted the protractor so that the centre point was aligned to the midsagittal plane. The data obtained from the sensors on this protractor were used to transform the tongue and lip data such that the *x,y,z* coordinate origin for the EMA data presented here is defined as a point directly posterior to the incisors in the sagittal plane (and parallel to the speaker's occlusal plane along this dimension), centred between the superior and inferior incisors in the axial plane and centred between the left and right incisors in the coronal plane.

High-fidelity acoustic data were collected using a Schoeps CMC6-U condenser microphone secured in a shock-mount stand. The microphone was positioned to the side of the mother, pointed towards her and placed at a distance of approximately 0.5 m. The microphone level was adjusted manually using a Behringer Eurorack UB802 microphone preamplifier at the beginning of the experiment in order to prevent amplitude clipping. The acoustic data were recorded at a sampling rate of 44.1 kHz and temporally synchronized with the EMA data automatically by the NDI Wave system during data collection.

### 2.3. Acoustic data analyses

The onset and offset of the vowel in each target word elicited in the audio recording were segmented manually in Praat [21] (see the electronic supplementary material, table S1 for the number of target vowels elicited for each register). The temporal midpoint of each vowel interval was calculated, and the acoustic and articulatory metrics described below were obtained at these vowel midpoint locations.

Formant values for the first four formants (F_1_–F_4_, in Hz) were extracted automatically in Praat using a custom-written script. In order to minimize formant-tracking errors, the automatic estimation parameters were optimized for each mother; the parameters that yielded formant tracks most closely approximating the visible formant trajectories in the broadband spectrograms were used for the automatic estimation for that particular mother. Additionally, F_1_ and F_2_ values were plotted and outlying items were checked manually via visual inspection of spectrograms and spectral slices at the vowel midpoints; accordingly, adjustments to formant values were made manually when necessary.

### 2.4. Electromagnetic articulography data analyses

EMA data were corrected for head movement and the origin of the axes was transformed as described above using custom-written Matlab [22] functions created and/or modified by Marke Tiede (Haskins Laboratories), Donald Derrick (New Zealand Institute of Language, Brain & Behaviour) and the second author. For qualitative midsagittal tongue and lip measures, *x*-values (posterior/anterior) and *z*-values (inferior/superior) were logged from the TB, TM, TT, lower lip and upper lip sensors. Second-order polynomial functions were fit to the three tongue sensors in order to estimate midsagittal tongue shape. Additionally, a quantitative metric was created using the *x*, *z*-values from the TM sensor, the sensor that most reliably captures the qualitative tongue differences evident in the polynomial curves. A triangle was fitted to the average speaker-normalized TM values for the three corner vowels in each context, and the area of this triangle was logged as the quantitative metric referred to as the ‘tongue triangle’. Although this metric provides a quantified articulatory comparison to the acoustic vowel triangle, the qualitative comparisons of the entire vocal tract shape are arguably more meaningful for the goals of the current study (see the electronic supplementary materials). For a sagittal lip measure, the average *x*-value for the upper and lower lip sensors was used as a metric corresponding to lip protrusion. For coronal lip measures, *y*-values (right/left) and *z*-values were logged from the four lip sensors. The area of a four-sided polygon fit to the average speaker-normalized values for the four lip sensors was calculated for each context and logged as the quantitative metric referred to as ‘lip aperture’. For this measure, large values indicate that the lips are spread, and small values indicate that the lips are closed and rounded.

### 2.5. Vocal tract length estimation

Vocal tract length was estimated using a combination of acoustic and articulatory data. Assuming a tube with uniform diameter throughout its length, the frequency (in Hz) of any given resonant formant *F_n_* (Hz) can be estimated using the formula

$$F_{n}=\frac{(2_{n}-1)c}{4L},$$

where *L* is the length of the tube (cm), *n* is the formant number and *c* is the speed of sound (cm s^−1^). In this model, the length of the tube is known but the formant frequencies are not. Conversely, given known formant frequencies, the length of the tube can be estimated. Accordingly, we first used values (in Hz) of F_1_–F_4_ to estimate the length of the uniform tube, which would be predicted to yield these resonances using the formula

$$L=\frac{c\sum_{n=1}^{m}n/2-0.25}{\sum_{n=1}^{m}F_{n}},$$

where *m* = 4 (number of formants) and *c* = 34 029 (approximated speed of sound in the atmosphere). As this model assumes a uniform tube, this estimate is most accurate for the vowel /ə/, which most closely approximates a tube without any constrictions. However, given that the vowels /i, u, a/ researched in the current study involve a vocal tract shape that significantly deviates from a uniform tube, modelling the vocal tract length using only the first two formants, which are very strongly dependent on vowel quality, would be inappropriate. In order to formulate a length estimate that is more reliable across vowel qualities, frequencies for formants F_3_ and F_4_ have been included in the estimate, because higher formants (above F_2_) tend to be more regularly spaced and deviate from those of a uniform tube to a lesser degree than do lower formants.

As this estimate represents the length of the entire vocal tract, any lengthening or shortening of the vocal tract due to protrusion or retraction of the lips would contribute to the estimate. We are not interested in the variation in vocal tract length due to lip protrusion or retraction, but rather to variations in the height of the larynx. Therefore, the average *x*-dimension values of the upper and lower lip EMA sensors (i.e. the horizontal position of the lip sensors in the sagittal plane) were subtracted from the above length estimate in order to remove the contribution of the lips to the overall vocal tract length. The result is an estimate of vocal tract length from the larynx to the EMA sensor origin, immediately posterior to the incisors. As this origin remains constant for a given speaker throughout the recording session, variation in this articulatory/acoustic vocal tract length estimate can be assumed to be due to variation in the height of the larynx.

## 3. Results

To account for individual variability in speech production and physical morphology, all values used for analyses were normalized by speaker. Separate linear mixed models (LME, using the *lme4* package) [23] in R [24] were constructed for each measure with condition (IDS, ADS and ES) as a within-subjects independent variable and speaker as a random effect (see table 1 for a summary of the results). Random slopes and intercepts were included for speaker in the LME models for lip aperture and vocal tract length; only random intercepts were included for speaker in the LME models for acoustic triangle and articulatory triangle as these models did not converge with the full random structure. As ADS was collected as the control measure for each mother, the results of each LME were followed by planned pairwise comparisons using Tukey tests to compare the IDS and ES conditions to ADS (see the electronic supplementary materials for the complete dataset used for these analyses).

**Table 1.** Results of linear mixed effect model analyses for vowel triangle, tongue triangle, lip protrusion and lip aperture as dependent variables, condition (IDS, ADS and ES) as the repeated-measures independent variable and speaker as a random effect.

		estimate (*β*)	s.e.	*t*
vowel triangle	(Intercept)	0.725	0.143	5.063
	ES	2.429	0.203	12*
	IDS	0.503	0.203	2.486*
tongue triangle	(Intercept)	0.4999	0.178	2.802
	ES	1.127	0.2271	4.961*
	IDS	0.453	0.2271	1.994
lip protrusion	(Intercept)	0.331	0.08	4.131
	ES	−1.129	0.109	−10.306*
	IDS	−0.103	0.089	−1.148
lip polygon (/a/)	(Intercept)	4.13	0.14	29.16
	ES	0.68	0.13	5.25*
	IDS	0.16	0.11	1.37
lip polygon (/i/)	(Intercept)	3.58	0.12	28.29
	ES	1.06	0.13	8.07*
	IDS	−0.12	0.11	−1.11
lip polygon (/u/)	(Intercept)	3.24	0.08	42.04
	ES	−0.43	0.07	−6.41*
	IDS	−0.05	0.05	−1.02
vocal tract length	(Intercept)	0.3	0.11	2.86
	ES	−0.22	0.15	−2.83*
	IDS	−0.53	0.15	−3.56*

*p < 0.05.

### 3.1. Hyper-acoustics in infant-directed speech

**Figure 1.**
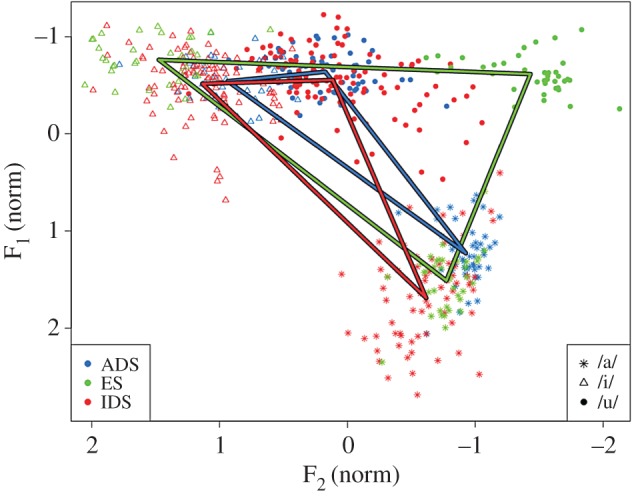
Speaker-normalized acoustic space for the ADS, IDS and ES conditions.

Figure 1 represents the acoustic vowel triangle areas for the three registers (IDS, ES and ADS) averaged across all speakers. As can be seen, the largest vowel triangle area was produced in the ES condition followed by IDS, then ADS. LME analyses (table 1) indicated that vowel triangle area for ES and IDS were each significantly different from ADS. Tukey pairwise comparisons confirmed that vowel triangle area is greater for ES than ADS (*z* = 12, *p* < 0.001), ES than IDS (*z* = 9.52, *p* < 0.001) and also IDS than ADS (*z* = 2.49, *p* = 0.035).

### 3.2. Tongue movements

**Figure 2.**
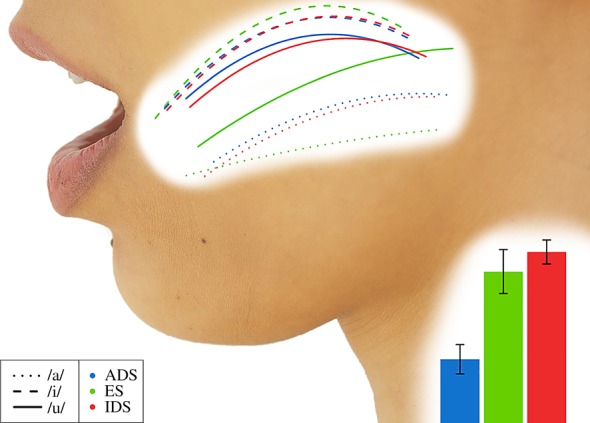
Average sagittal tongue shapes and larynx height estimates.

Figure 2 illustrates the average tongue shape created by fitting polynomial curves to the values of the three tongue sensors in the midsagittal plane for the production of the three vowels in each register. The articulatory configurations for ES were used to establish a prototype of ES to determine whether these configurations differ from ADS and whether IDS is exaggerated in a similar manner (see the electronic supplementary materials). In order to provide a direct articulatory metric to compare with the acoustic triangle metric, the TM (tongue mid) tongue triangle measure was used in analyses. LME results and follow-up Tukey tests indicated that there were significant differences in tongue triangle sizes (table 1): ES > ADS (*z* = 4.96, *p* < 0.001) and ES > IDS (*z* = 2.97, *p* = 0.008), but there was no significant difference between IDS and ADS (*z* = 1.99, *p* = 0.114).

### 3.3. Lip movements (protrusion and aperture)

**Figure 3.**
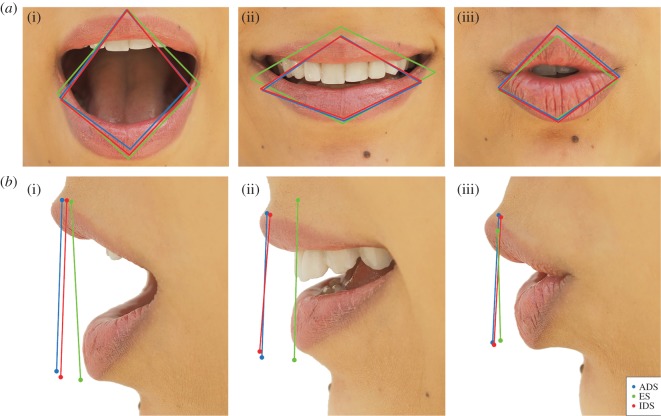
Average lip shapes in the coronal (*a*(i)–(iii)) and sagittal (*b*(i)–(iii)) dimensions for /a/ (*a*(i),*b*(i)), /i/ (*a*(ii),*b*(ii)), and /u/ (*a*(iii),*b*(iii)).

Figure 3 illustrates the average lip shape in the midsagittal (lip protrusion) and coronal planes (lip aperture) for the three vowels in each condition. LME results indicated that there were significant differences in both lip protrusion and lip aperture (table 1) between ES and ADS, but not between IDS and ADS. Specifically, speakers' degree of lip protrusion differed significantly between ES and ADS conditions (*z* = −10.31, *p* = 0.001) and between IDS and ES (*z* = 10.59, *p* < 0.001), but not between IDS and ADS (*z* = 1.148, *p* = 0.482). Similar results were observed for the analyses for lip aperture that were conducted separately for each vowel: lip aperture was more exaggerated in ES than in ADS (/a/ *z* = 5.24, *p* < 0.001; /i/ *z* = 8.07, *p* < 0.001; /u/ *z* = −6.41, *p* = 0.001), in IDS than in ES (/a/ *z* = 4.39, *p* < 0.001; /i/ *z* = 10.89, *p* < 0.001; /u/ *z* = 6.27, *p* < 0.001), but there were no significant differences between IDS and ADS (/a/ *z* = 1.37, *p* = 0.353; /i/ *z* = 1. 11, *p* = 0.504; /u/ *z* = −1.02, *p* = 0.558).

The results for these two articulatory loci suggest that, while very minor articulatory adjustments may be observed for IDS compared to ADS, there is no evidence to suggest that IDS vowels are truly hyperarticulated variants of these vowels. Moreover, it is clear that the observed articulatory modifications of the tongue (figure 2) and lips (figure 3) cannot account for the acoustic differences found between IDS and ADS (figure 1).

### 3.4. Vocal tract

**Figure 4.**
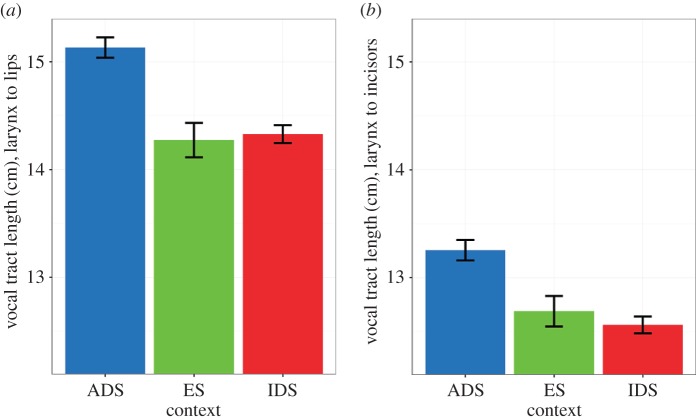
Vocal tract length estimates for ADS, ES and IDS normalized by speaker and converted to cm using overall mean and standard deviation ((*a*) total vocal tract length from larynx to lips; (*b*) vocal tract length from larynx to incisors; error bars represent s.e.m.).

An estimate of the length of the vocal tract from the larynx to the incisors was used as the dependent variable in LME models. Figure 4*a* displays the total estimated vocal tract length, i.e. from the larynx to the lips. The range of values in ADS was fairly consistent with observed measures of average vocal tract length for similar populations [25] (*M* length = 15.82 cm, s.d. = 1.63). Figure 4*b* displays the length estimates from larynx to incisors that were used in the statistical analysis here. The LME model results (table 1) showed that vocal tract length was significantly different for ES and ADS (*z* = −2.83, *p* = 0.013), but in contrast to the tongue and lip articulatory adjustments, there was no difference in vocal tract length between IDS and ES (*z* = −0.734, *p* = 0.743). Most importantly, the vocal tract length was significantly shorter in IDS than in ADS (*z* = −3.56, *p* = 0.001). Crucially, these vocal tract length differences do not include any contribution of the lips to the overall length; thus, as the lip end of the vocal tract estimate is fixed, the observed differences can only be attributed to variation in the height of the larynx.

The results show that the sole articulatory modulation that reliably distinguishes IDS from ADS is the length of the vocal tract; IDS and ADS do not differ in tongue movements, lip protrusion or lip aperture. As the vocal tract length estimate used in the current study excludes any contribution of the lips, these results also suggest that the reduced vocal tract length in IDS is a result of a raised larynx. When the larynx is raised and the vocal folds are tightened, F_0_ also increases as does the frequency and separation of all formants. To test whether it is indeed the shortening of the vocal tract by raising of the larynx that relates to hyper-acoustic vowels rather than tongue articulations or lip movements, we correlated the size of the acoustic triangle (figure 1) with F_0_, tongue triangle size, vocal tract length, lip aperture and lip protrusion for each register, IDS, ES and ADS. IDS acoustic vowel triangle size was significantly correlated with F_0_ and vocal tract length but with no other articulatory measure (table 2), supporting the premise that vocal tract shortening is due to laryngeal raising.

**Table 2.** Results of correlation analyses (Pearson's *r*) between acoustic vowel triangle size, F_0_ and articulatory triangle, vocal tract length, lip aperture (for vowels /a,i,u/), lip protrusion for ADS, ES and IDS, respectively.

acoustic vowel triangle	tongue triangle	vocal tract length	lip aperture /a/	lip aperture /i/	lip aperture /u/	lip protrusion	F_0_
ADS	0.688	0.375	0.464	0.287	0.033	0.215	−0.351
ES	−0.609	−0.457	−0.692	−0.311	0.061	0.760*	0.605
IDS	0.338	−0.801*	0.037	−0.193	0.164	0.628	0.796*

*p < 0.05.

## 4. Discussion

Mothers produced significantly larger acoustic vowel triangles in IDS than in ADS, thus showing, along with many previous studies (e.g. [5,6]), the presence of hyper-acoustics in speech addressed to young infants. Three articulatory loci for this hyper-acoustic IDS effect were investigated: tongue movements, lip movements and vocal tract length. The results showed that the tongue and lip articulatory movements did not differ between IDS and ADS, but mothers’ vocal tract was shorter in IDS than in ADS. Moreover, the only significant correlations for IDS were between acoustic vowel triangle area and vocal tract length, and between acoustic vowel triangle area and mean pitch (F_0_). Thus, the well-established phenomenon of hyper-acoustic vowels in IDS compared with ADS vowels is *solely* due to shortening of the vocal tract by raising the larynx, a strategy that is thought to be used to signal non-threatening intentions.

According to the linguistic function hypothesis, the hyper-acoustic effect of larger vowel space in IDS [5,6] is a product of parents' unconscious exaggerated articulations directed at facilitating language acquisition [26]. However, all previous evidence for this hypothesis has been based only on acoustic measures of parental speech, with no articulatory measures being included. Without articulatory evidence, the term ‘hyperarticulation’ is a misnomer. Such articulatory evidence is provided here and there is no evidence of hyper*articulation* of tongue and lip movements in IDS, despite evidence of hyper-*acoustics* in IDS.

Our results instead suggest that the occurrence of hyper-acoustics in IDS is a side effect of shortening the vocal tract. The vocal tract can be shortened by retracting the lips and/or by raising the larynx. So (i) the expected acoustic differences between IDS and ADS were observed and (ii) there were no lingual or labial articulatory differences between IDS and ADS productions and lip retraction did not occur in IDS, but (iii) vocal tract length from the larynx to the incisors *excluding the contribution of the lips* is shorter for IDS than ADS and (iv) of the three articulatory measures, only vowel triangle area was correlated with vocal tract length. Accordingly, we propose that mothers shorten their vocal tract length by raising their larynx in IDS, resulting in higher pitch and greater acoustic vowel triangle area via more extreme, more separated F_1_ and F_2_.

Heightened pitch and exaggerated pitch contours have been postulated as primordial qualities of IDS used by parents for expressing vocal emotion and emotionally regulating their infants [3,27]. It has also been proposed that heightened pitch and increased formant frequencies are used by humans and other species to appear smaller and less threatening [28,29], whereas decreased pitch and formant frequencies are used to appear larger and convey aggressiveness [30–34].

Heightened pitch and exaggerated pitch contours have also been postulated as the phylogenetic antecedents of IDS. It has been suggested that in pre-linguistic communities there was pressure for mothers to leave their infants on the ground in order to forage for food, and high-pitched vocalizations were used to replace physical proximity to comfort, soothe and maintain the attention of their infants [35]. The same development appears to occur across ontogeny; as very young infants are unable to decode the linguistic messages in speech, the core initial function of IDS appears to be conveying a non-threatening attitude, expressing positive emotions and focusing attention on the communicative process through prosodic speech patterns [26,36]. These prosodic speech patterns (i) arouse infant behavioural and neural responses to incoming speech, giving IDS an advantage in early linguistic processing [37–39] and (ii) are associated with positive emotion in IDS and the qualities that underlie infants' early preferences for IDS [2,40–42]. Additionally, exaggerated pitch and pitch patterns facilitate infants’ successful segmentation of the incoming speech stream [43] and their discrimination of phonetic categories [44]. So the hyper-acoustic vowel space in IDS might be a vestige of parents (unconsciously) maintaining infant attention and a non-threatening attitude while physically separated from the infant, rather than an (unconscious) desire to teach about vowel space.

The reduction in vocal tract length also results in greater acoustic similarity between maternal vocalizations and infants' own early vowel productions (i.e. speech sounds produced by a smaller, infant speaker). This similarity to their own speech has been proposed to drive infants’ preferences for IDS; infants prefer to listen to speech with the acoustic and linguistic qualities of their own vocalizations compared to speech produced by an adult [45,46]. Hence, infant-directed vocalizations exhibiting the qualities of a shorter vocal tract may also be a primitive social or emotional vocal convergence, an alignment in interlocutors' speech qualities during their communicative interactions [47,48]. Mothers’ unconscious efforts to approximate their infants' vocalizations during the early mother–infant contingent interactions can also reflect maternal sensitivity to the communicative cues from their infants. The sensitivity that a mother develops towards her infant in the first days and months of life, in turn, strengthens her infant's early communicative, social-cognitive and linguistic development [49].

These findings provide the first evidence that hyper-acoustic vowels in IDS compared to ADS are a product of the articulatory adjustment of laryngeal raising. It is noteworthy that this adjustment has only been detected in the specific population of monolingual Australian English mothers addressing 11-month-old infants. This highlights a limitation of the present experiment, but opens an intriguing question about the potential interplay between mothers' use of this articulatory adjustment and factors endogenous and exogenous to the mother–infant dyad that impact the presence of hyper-acoustic vowels in IDS. Such factors include infants’ sensory [13,14] and cognitive abilities [15], the acoustic and prosodic characteristics of the vowels considered in the analyses [50–52], and the language used in the interaction [18,53,54]. These variations in the presence of acoustically exaggerated vowels across different communicative situations and infant populations are consistent with the possibility that mothers employ different articulatory adjustments to emphasize certain linguistic or emotional qualities in their speech that are most appropriate for particular infants' cognitive, linguistic or emotional needs.

The present findings must not be interpreted as evidence that hyper-acoustics in IDS do not serve a linguistic function. The results here suggest that parents do not exaggerate the production of their vowels in order unconsciously to teach their infants about language; rather they shorten their vocal tract unconsciously to appear smaller and less threatening and thereby express emotion, and arouse and maintain attention. Nevertheless, the action of raising the larynx concurrently raises and separates formant frequencies, resulting in more distinct vowel categories that are easier to discriminate and reproduce, which may assist infants in language acquisition processes such as speech perception [55] and lexical processing [56]. So hyper-acoustics in IDS may be an evolutionary accident, but if so, it is a happy accident. In evolution, IDS may well have emerged as a tool for pre-linguistic hominid mothers to appear small and non-threatening [30–34], and/or comfort and soothe their infants and regulate infant affect [35]. Only once human language emerged, did the increased vowel space in this proto-IDS come to acquire a purpose—to facilitate language acquisition in infants’ first months and years of life.

## Supplementary Material

Table S1

## Supplementary Material

Articulatory configurations

## Supplementary Material

Dataset

## Footnotes

1 Raising the larynx is not the only way to create tension in the vocal folds. The vocal folds can also be stretched via contraction of the cricothyroid muscles, which can occur independently of changes in laryngeal height. However, unlike contraction of the cricothyroid muscles, tightening the vocal folds by raising the larynx has the by-product of increasing the values of all formants as well as separating the distance between formants (including F_1_ and F_2_, which are used to measure the vowel triangle in IDS). Thus, while both cricothyroid contraction and laryngeal raising (resulting in cricoid cartilage rotation) tighten the vocal folds, only the action of raising the larynx also changes the formant structure.

## Data accessibility

Refer to the electronic supplementary material for the complete data file used for all the analyses reported in this manuscript.
