# Supplementary material for: The origins of babytalk: smiling, teaching or social convergence?
Source: R Soc Open Sci. 2017 Aug 2;4(8):170306. doi: 10.1098/rsos.170306 (PMC5579095; doi:10.1098/rsos.170306)
Supplement: Table S1 [file rsos170306supp1.docx]

Table S1. Number of vowel tokens elicited from each participant in the infant-directed speech (IDS), adult-directed speech (ADS), and exaggerated speech (ES) conditions.

| Participant | IDS | ADS | ES |
| --- | --- | --- | --- |
| Speaker 1 | 46 | 24 | 12 |
| Speaker 2 | 47 | 17 | 14 |
| Speaker 3 | 38 | 13 | 15 |
| Speaker 4 | 37 | 36 | 14 |
| Speaker 5 | 23 | 13 | 15 |
| Speaker 6 | 34 | 10 | 15 |
| Speaker 7 | 27 | 18 | 15 |
| Speaker 8 | 32 | 15 | 15 |
| **Total: 545** | 284 | 146 | 115 |
