# Supplementary material for: The origins of babytalk: smiling, teaching or social convergence?
Source: R Soc Open Sci. 2017 Aug 2;4(8):170306. doi: 10.1098/rsos.170306 (PMC5579095; doi:10.1098/rsos.170306)
Supplement: Articulatory configurations [file rsos170306supp2.docx]

**Articulatory Configurations**

In order to establish how exaggerated, truly hyperarticulated speech differs from regular speech, ADS, we first compared the articulatory configurations for ES vs. ADS for each vowel to provide a prototype of hyperarticulated speech, to be used to qualitatively determine whether IDS is hyperarticulated in a similar manner. Qualitative analysis includes speaker-normalised upper and lower lip position averaged for each context, as well as second-order polynomial curves fitted to the speaker-normalised values for the tongue sensors, averaged for each context. This allows for qualitative visual comparison of how these portions of the vocal tract, as a whole, differ between the ADS, IDS, and ES contexts. This resulted in the following characteristics of hyperarticulated vowels:

- /a/: the tongue is lowered and slightly fronted; the lips are wider and more retracted,
- /i/: the tongue is higher; the lips are wider and more retracted, and
- /u/: the body of the tongue is lowered and retracted, with the back of the tongue raised toward the back of the mouth; the lips are closer together.

Using these prototypical configurations for exaggerated, hyperarticulated vowel productions, we qualitatively compared IDS to ADS to determine if IDS vowels are truly hyperarticulated in the same manner. Although some minor articulatory differences can be observed for IDS compared to ADS (e.g., the front of the tongue is very slightly lowered for /a/ and /u/ in IDS, the lips are very slightly retracted for /a/ in IDS), it is clear that the articulation of IDS vowels is much closer to ADS than to ES. Furthermore, these minor articulatory differences cannot account for the acoustic distinctions observed in Figure 1 (main text). For example, IDS /a/ is realised with the largest acoustic difference compared to ADS, yet ES /a/ is realised with the largest articulatory difference compared to ADS, and for /i/, there is no difference in the shape of the tongue or the lips compared to ADS, yet F2 is raised for IDS vs. ADS /i/.
